# Renin-Angiotensin System Inhibitors in COVID-19: Current Concepts

**DOI:** 10.1155/2020/1025913

**Published:** 2020-10-21

**Authors:** Kunal Mahajan, Prakash Chand Negi, Neeraj Ganju, Sachin Sondhi, Naresh Gaur, Rao Somendra

**Affiliations:** ^1^Department of Cardiology, Indira Gandhi Medical College, Shimla 171001, India; ^2^ACE Heart and Vascular Institute, Mohali, Punjab 160062, India

## Abstract

The functional receptor to SARS-CoV-2, the virus responsible for the coronavirus disease 2019 (COVID-19) pandemic, is angiotensin-converting enzyme-2 (ACE-2), the same enzyme that physiologically counters the renin-angiotensin system (RAS) activation. Some researchers have questioned RAS inhibitors' safety in COVID-19 patients since these drugs have demonstrated an increase in ACE-2 expression in preclinical studies; therefore, they may facilitate viral invasion. On the contrary, others have hypothesized a protective role of RAS inhibitors against COVID-19-associated lung injury. Overall, the data are grossly inadequate to reach any conclusion since no human trials have yet evaluated the effects of RAS inhibitors in COVID-19. We review the current data and pathophysiological mechanisms behind this intriguing interplay between the RAS inhibitors and the COVID-19.

## 1. Introduction

The whole world is witnessing a prodigious attack on people's healthcare and lives by a novel coronavirus named severe acute respiratory syndrome coronavirus-2 (SARS-CoV-2) by the World Health Organization. The disease which it causes was named coronavirus disease 2019 (COVID-19). [1] Since the first case that broke out in Wuhan, China, in December 2019, this virus has infected more than 16 million people across the globe and has caused more than 6,00,000 deaths. Evidence suggests that hypertension (HTN), diabetes mellitus (DM), coronary artery disease (CAD), and chronic heart failure (CHF) are among the commonly associated comorbidities with COVID-19 [1]. In a retrospective analysis of 416 hospitalized COVID patients from Wuhan, HTN, DM, CAD, and CHF were seen in 30.5%, 14.4%, 10.6%, and 4.1% of patients, respectively [2]. The analysis of the largest available dataset of COVID-19-positive patients till date involving 44,672 patients reveals that the case fatality rate was higher for patients with HTN (6%), DM (7.3%), and prior cardiovascular disease (10.5%), when compared to the overall study population (2.3%) [3]. Similar trends of increased mortality with comorbidities in COVID-19 patients have been observed in other studies also [2, 4]. Many patients with the abovementioned comorbidities are prescribed treatment with renin-angiotensin system (RAS) inhibitors, such as angiotensin-converting enzyme inhibitors (ACE-I) or angiotensin receptor blockers (ARB). Of late, there has been much speculation regarding the use of RAS inhibitors in patients with COVID-19 [5]. This is because SARS-CoV-2 enters the human body via ACE-2 (angiotensin-converting enzyme-2) receptors, which might be overexpressed in patients who are on ACE-I/ARB [5]. Nevertheless, evidence-based medicine strongly recommends the use of ACE-I/ARB in patients with HTN, DM, postmyocardial infarction, and chronic heart failure. Consequently, the controversy exists regarding the use of ACE-I/ARB in patients at risk for, being evaluated for, or with COVID-19.

## 2. Context-Specific Basics of the Renin-Angiotensin System and ACE-2

**Figure 1:**
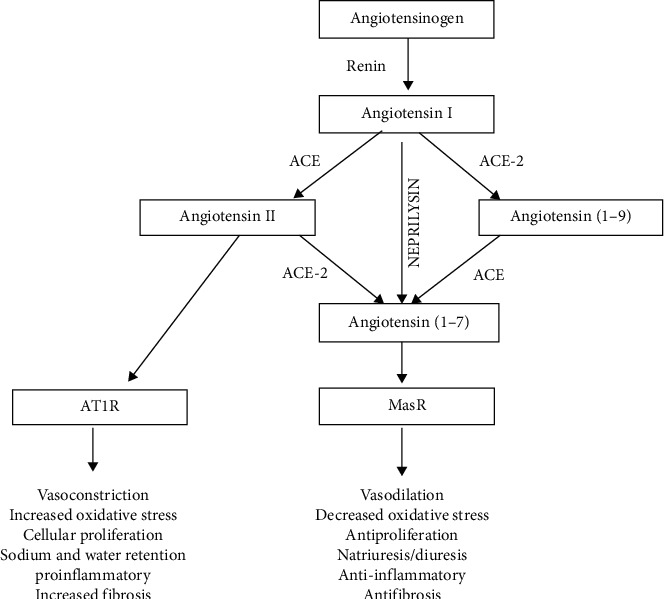
Roles of ACE (angiotensin-converting enzyme) and ACE-2 (angiotensin-converting enzyme-2) in the renin-angiotensin system. Renin cleaves angiotensinogen produced by the liver to angiotensin I which is further cleaved to angiotensin II by ACE enzyme. The primary function of the ACE-2 enzyme is to cleave angiotensin II to angiotensin (1–7) and angiotensin I to inactive angiotensin (1–9). The angiotensin II exerts its negative effects of vasoconstriction, proinflammation, and profibrosis through its interaction with the AT1R (angiotensin II type 1 receptor). In contrast, the angiotensin (1–7) exerts its beneficial effects of vasodilation, anti-inflammation, and antifibrosis through its interaction with MasR (Mas receptor).

Activation of RAS is one of the most important mechanisms responsible for local and systemic vascular remodeling, endothelial cell dysfunction, and hypertension [6]. Renin is a protease protein secreted solely by the juxtaglomerular cells in the kidneys. Plasma renin then converts angiotensinogen (produced by the liver) to angiotensin I (Ang-I), which is subsequently converted to angiotensin II (Ang-II) by the angiotensin-converting enzyme (ACE). Ang-II interacts with G protein-coupled Ang-II type 1 (AT1) receptors to activate numerous cellular processes, including aldosterone production, vasoconstriction, vascular inflammation, sympathetic activation, and endothelial dysfunction, ROS (reactive oxygen species) generation, and cardiac/vascular remodeling [5, 6]. All these processes contribute to causing vascular damage, hypertension, and accelerate hypertensive end-organ damage [6] (Figure 1).

ACE-2 is a homolog of ACE, and its principal function is to cleave angiotensin I to inactive angiotensin (1–9) and counterregulation of the harmful effects of angiotensin II by degrading it to angiotensin (1–7) [6]. Angiotensin (1–7) further interacts with the Mas receptor (MasR) and exerts its protective effects in vasodilation, reduction in oxidative stress, inflammatory stress, and mitigation of vascular/tissue remodeling in various organs, principally the heart, kidneys, lungs, and liver. Thus, the “ACE-2-angiotensin (1–7)-Mas axis” counteracts the effects of the ACE-Ang-II-AT1 axis” (Figure 1) [6]. Therefore, any agent that augments the level/activity of ACE-2 is likely to have protective effects by decreasing Ang-II and increasing angiotensin (1–7) [5, 6].

**Figure 2:**
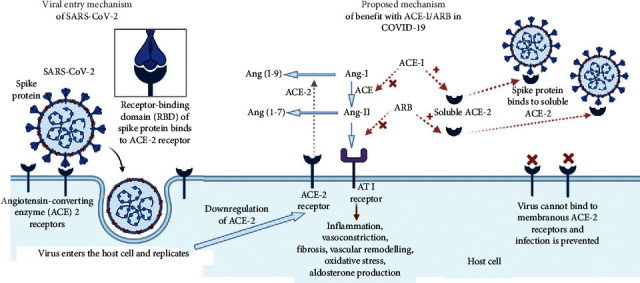
The interplay between RAS (renin-angiotensin system) and COVID-19 (coronavirus disease 2019). SARS-CoV-2 (severe acute respiratory syndrome coronavirus-2) enters the host epithelial cell after its spike protein's receptor-binding domain binds to the membrane-bound ACE-2 (angiotensin-converting enzyme-2) receptor. The cellular entry is followed by endocytosis and further viral replication and subsequent release. SARS-CoV-2, after entry inside the cell, causes downregulation of further ACE-2 expression. ACE-2 is responsible for the conversion of Ang (angiotensin) II to Ang (1–7) and Ang-I to inactive Ang (1–9). The downregulation of ACE-2 results in an unopposed action of Ang-II through its AT1 (angiotensin II type 1) receptor. ACE-I (angiotensin-converting enzyme inhibitor) and ARB (angiotensin receptor blocker) oppose Ang-II's action by blocking its synthesis and receptor, respectively. ACE-I/ARB may also cause overexpression of the soluble form of ACE-2, which intercepts the binding of SARS-CoV-2 with membrane-bound ACE-2, thus inhibiting the viral entry into the host cell.

Functionally, there are two forms of ACE-2 in the human body: the soluble (circulating) form and the tissue-bound (membranous) form [6, 7]. Usually, the soluble form circulates in small amounts in the blood. On the other hand, the membranous form is widely expressed in organs, such as the lungs, heart, kidneys, and gastrointestinal tract. The functional role of ACE-2 in the lungs appears to be relatively minimal under normal conditions. However, it may be upregulated in certain clinical states like SARS-CoV-2 infection. In the context of coronavirus infection, the interest lies mainly in the tissue-bound ACE-2, which contains a structural transmembrane domain that anchors its extracellular receptor domain to the plasma membrane. The extracellular domain of membranous ACE-2 has a receptor for the spike (S) protein of SARS-CoV-2, which provides attachment for the virus to enter the cell (Figure 2). The other form of ACE-2, i.e., the soluble form, lacks the transmembrane anchor and is free to circulate in the blood [6, 7]. Interestingly, the soluble form has been shown to inhibit viral replication in animal studies, possibly by acting as a competitive interceptor of SARS-CoV-2 since binding to the soluble form prevents the binding of the spike protein of SARS-CoV-2 to the membranous form of ACE-2 [7].

## 3. Effect of ACE-I/ARB on ACE-2

There are conflicting data regarding the effect of ACE-Is and ARBs on the level/activity of ACE-2 [8–13]. Both ACE-Is and ARBs have different effects on Ang-II, with the former inhibiting its formation (decrease in levels of Ang-II levels) while the latter blocking its receptor (no effect on levels of Ang-II). Since Ang-II is the primary substrate for ACE-2, the effects of ACE-I/ARB on the levels of ACE-2 are likely to be different [6]. Expectedly, animal studies have shown inconsistent results regarding the expression of ACE-2 in patients treated with ACE-I/ARB. Few animal studies have shown an increased expression [8–10], but others have shown no effect [11]. Human studies have also revealed inconsistent findings [12–14]. A study showed increased intestinal mRNA levels of ACE-2 in patients who were on treatment with ACE-Is [12]. In a study involving 859 patients with type 1 DM, the levels of ACE-2 were increased among patients who were on ACE-I therapy [13]. In a study evaluating antihypertensive agents' role on urinary ACE-2 levels among patients with HTN, elevated levels of urinary ACE-2 were seen in patients treated with ARB olmesartan.

In contrast, no effect was seen with ACE-I enalapril and other ARBs such as losartan, candesartan, valsartan, and telmisartan [14]. Furthermore, various negative studies have not shown elevation in the levels of ACE-2 in patients on treatment with ACE-I/ARB [15, 16]. Therefore, the available data are grossly inconsistent to make any judgments about the change in the level/activity of ACE-2 in patients on RAS inhibitors. Importantly, no evidence exists to date, demonstrating increased expression of ACE-2 in the lung tissue with the use of ACE-I/ARB. Furthermore, it is yet not clear whether this increased expression would indeed facilitate the entry of SARS-CoV-2 inside the alveolar epithelial cells.

## 4. ACE-I/ARB in COVID-19

Few studies have shown increased levels of ACE-2 with ACE-I/ARB, resulting in a theoretical possibility that the increased ACE-2 may facilitate the invasion by SARS-CoV-2 and potentiate the severity of organ damage caused by this novel coronavirus. The concern has been further provoked by the exaggerated news and media coverage, which has resulted in sudden discontinuation of ACE-I/ARB treatment by many patients. However, in a study involving 187 hospitalized COVID patients by Guo et al. [4], the mortality rates of patients with and without the use of ACE-I/ARB were 36.8% (7 of 19) and 25.6% (43 of 168), respectively. Unfortunately, the number of patients who were on ACE-I/ARB therapy was too small (19/187) to reach any conclusion [4]. Similarly, few other observational studies have failed to demonstrate any association between the use of ACE-I/ARB and increased risk and severity of infection with SARS-CoV-2 [17–19].

On the contrary, some researchers have a different perspective and suggest a protective effect of ACE-I/ARB in COVID patients [6, 20]. There is some evidence that ACE-I/ARB may be beneficial in patients with acute lung injury or acute respiratory distress syndrome (ARDS), induced by some influenza strains and SARS-CoV. [21–23] In a retrospective case-control study involving 182 patients with ARDS, the use of ACE-I/ARB was associated with a reduction in mortality rates [24]. Similarly, in a meta-analysis of 37 studies, ACE-Is were associated with reduced risk of pneumonia and pneumonia-related mortality compared with control treatment [21]. However, there are yet no data to show a similar benefit to COVID-19 patients. The proposed protective mechanism is based on the downregulation of ACE-2 by SARS-CoV-2 (Figure 2). After the initial entry of SARS-CoV-2 into the cells via ACE-2, the virus further downregulates the ACE-2 expression, thereby mitigating the enzyme's protective effects [6]. As a result, the activity of Ang-II is augmented and leads to local RAS activation, resulting in the organ damage seen with COVID-19 [6]. ACE-I/ARB will protect against this organ damage by (1) possible upregulation of ACE-2 activity, (2) reduced levels of Ang-II (by ACE-Is), and (3) blocking the ATI receptor of Ang-II (by ARBs). The above mechanistic hypothesis is further supported by a small study of 12 COVID-19 patients with pneumonia/ARDS, which showed that angiotensin II levels in the plasma sample from these patients were markedly elevated and linearly associated with viral load and lung injury [25]. Another postulated mechanism of benefit is the ACE-I/ARB-induced increase in the level of the soluble form of ACE-2, which may act as a competitive interceptor of SARS-CoV-2 and slow virus entry into the cells and protect from lung injury [7] (Figure 2).

The use of ARNI (angiotensin receptor neprilysin inhibitor) in COVID-19 patients is likely to exhibit the same responses (beneficial/harmful) as ACE/ARB. Interestingly, an author recently suggested an additional mechanism of benefit with ARNI in COVID-19 [26]. Based on its studies in acute heart failure patients, sacubitril/valsartan demonstrated a reduction in the concentration of proinflammatory cytokines and neutrophil count while increasing the lymphocyte count more than valsartan alone or placebo. Such an effect is likely to protect against the COVID-19-associated organ damage, which is usually mediated by cytokine storm and decreased lymphocyte count [26]. Secondly, ARNI has demonstrated a stabilizing effect among patients admitted with acute heart failure and decreased the composite end point of all-cause death and heart failure hospitalizations [27]. The benefit was attributable to a significant reduction in the NT-pro-BNP levels up to 50% achieved within the first week after initiation [27]. Thus, the use of ARNI becomes exciting in the context of COVID-19 since a substantial proportion of severe COVID-19 patients develop acute cardiac injury and develop signs and symptoms of heart failure [28]. NT-pro-BNP is often elevated in patients with severe COVID-19 regardless of the left ventricular dysfunction and is associated with worse outcomes [29, 30]. Thus, the use of ARNI in patients with COVID-19 is likely to benefit by causing a decrease in NT-pro-BNP levels and maximizing the anti-inflammatory effects of an enhanced natriuretic peptide system and contain the effects of angiotensin II [30]. However, it is again emphasized that all proposed beneficial mechanisms are anecdotal, and no clinical data are yet available to recommend the initiation of ACE-I/ARB or ARNI to treat patients with COVID-19-associated lung injury. Importantly, trials are being conducted to test the efficacy of losartan as a treatment for COVID-19 patients who have not previously received treatment with a RAS inhibitor (NCT04312009 and NCT04311177) [6].

## 5. Recommendations for the Use of ACE-I/ARB in the COVID-19 Era

As aforementioned, the patients with comorbidities such as HTN, DM, CAD, and heart failure are the ones to develop severe disease and with the highest mortality because of COVID-19 [2–4, 31]. Majority of the patients with these comorbidities are on RAS blockade with ACE-I/ARB, and there is abundant and substantial evidence of the mortality-lowering effects of RAS inhibitors in cardiovascular disease [6, 31, 32]. Abrupt discontinuation of therapy puts them at risk of complications/rebound exacerbations of the disease. In patients with HTN, discontinuation/switching of a drug can lead to rebound hypertension and transient suboptimal control with the new agent, thus predisposing the patient to intermittent high blood pressure fluctuations, which can augment the risk of developing cardiovascular events [32]. Similarly, in heart failure patients, sudden discontinuation of ACE-Is has been shown to result in a deteriorating clinical status with a possible relative increase in mortality [31]. In postmyocardial infarction patients with left ventricular dysfunction, the RAS blockade provides mortality benefit [32]. Similarly, the use of ACE-I/ARB reduces the risk of diabetic complications in patients with DM. Until more data become available, we feel that continuation of ACE-I/ARB may or may not increase susceptibility to SARS-CoV-2 infection, but sudden discontinuation of ACE-I/ARB is more likely to result in decompensation of previously stable clinical state. Therefore, we recommend the following regarding the use of ACE-I/ARB in COVID-19 patients:

1. All patients previously using ACE-I/ARB should continue taking them irrespective of the COVID status.
2. If required, discontinuation should be on clinical grounds (e.g., development of hyperkalemia, worsening renal functions, or hypotension), but should not be directed by COVID status.
3. Initiation of use of ACE-I/ARB in patients newly diagnosed with HTN, DM, and heart failure should be done according to the clinical recommendation and standard guidelines, and not according to their COVID status.
4. While initiating, the decision should be taken on a case-to-case basis. For example, among a newly diagnosed patient with hypertension but no diabetes/end-organ damage, other antihypertensive agents may be tried first. On the contrary, if hypertension is accompanied by diabetes and end-organ damage like proteinuria, it is preferable to start ACE-I/ARB.
5. ACE-I/ARB should not be started per se to treat COVID-19 (in the absence of their guideline-recommended indications such as DM, HTN, and heart failure).
6. Similar recommendations should be used for ARNI also.

The above recommendations are based on the advisories released by various national/international scientific societies of repute [1, 5].

## 6. Conclusion

Cardiovascular comorbidities are commonly associated with increased severity and complications in COVID-19. Guidelines recommend the use of ACE-I/ARB in patients with cardiovascular comorbidities, but there is speculation regarding an increased risk of COVID-19 infection and its severity with the use of these drugs. To add to the confusion, some researchers have suggested a beneficial effect of ACE-I/ARB in preventing COVID-19-associated organ damage. Therefore, to use them or not is like a double-edged sword for the treating physician. The lack of evidence from observational studies further aggravates the dilemma. We need focused prospective studies and randomized trials to ascertain the real association and impact of ACE-I/ARB use in patients with COVID-19. Based on this literature review, and until further data are available, we recommend that RAS inhibitors be continued in patients in otherwise stable conditions who are at risk for, being evaluated for, or with COVID-19.

## Data Availability

The data used to support the findings of the study are available from the corresponding author upon request.
